# 502. Telemedicine Service for Evaluation of Pediatric Outpatients at High Risk for Severe COVID-19

**DOI:** 10.1093/ofid/ofad500.571

**Published:** 2023-11-27

**Authors:** Kathryn E Weakley, Kristina Bryant

**Affiliations:** Norton Children's and University of Louisville School of Medicine, Louisville, Kentucky; University of Louisville and Norton Children's, Louisville, KY

## Abstract

**Background:**

During the COVID-19 pandemic, neutralizing monoclonal antibody (mAb) products received emergency use authorization for treatment of mild to moderate COVID-19 in high-risk patients. Expert consensus guidance recommended individualized risk assessment when considering mAb therapies in children and adolescents, as well as a process to ensure safe and timely administration. In one health system, high demand for mAb to treat adult patients, a finite supply, and a lower risk of severe COVID-19 in children led to allocation of a limited number of doses of mAb for pediatric use. The clinical outcomes of pediatric patients evaluated for allocation of mAb using telemedicine have not been well-described.

**Methods:**

A pediatric infectious diseases (PID) telemedicine service was established to evaluate children with COVID-19 and adjudicate the administration of mAb based on risk assessment, symptoms, physical exam, and the availability of mAb product. Only PID providers were permitted to order mAb in pediatric patients. Demographics and clinical outcomes data were retrospectively collected from the charts of patients evaluated between August 1, 2021 and April 30, 2022.

**Results:**

Two hundred sixty-seven patients were evaluated; 134 medical records have been reviewed to date. The average age was 14.7 years. The most common high-risk condition was obesity. The median time from onset of symptoms to telemedicine evaluation was four days. The median time from COVID-19 diagnosis to telemedicine evaluation was 1.5 days. Six patients were acutely referred to the emergency department (ED); three were admitted, one was given mAb and discharged, and two did not report to the ED as advised. Outpatient mAb was recommended for 12 patients; one declined and 11 received therapy with no serious adverse events. Only two of the remaining 116 untreated patients required COVID-related ED visits within 14 days of telemedicine evaluation; one was admitted with idiopathic thrombocytopenia and the other was discharged. All 134 patients made a full recovery.

**Figure:**
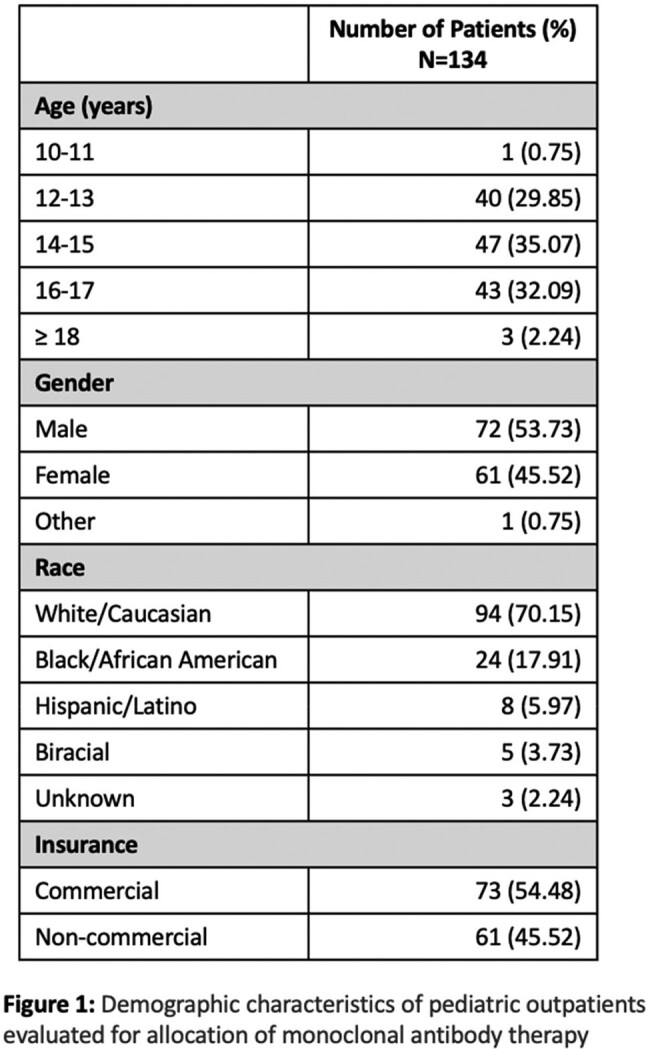

**Figure:**
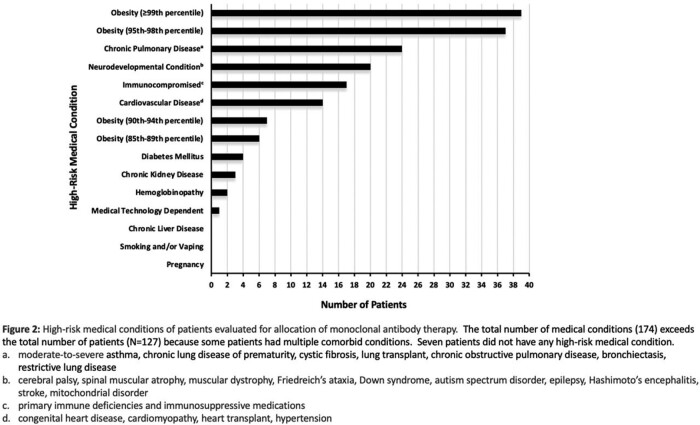

**Conclusion:**

Most children evaluated for mAb did not receive treatment and recovered. Telemedicine is a safe and effective way to evaluate patients with COVID-19. Individualized risk assessment can assist with allocation of scarce resources such as mAb.

**Disclosures:**

**Kathryn E. Weakley, MD, MSc**, Pfizer: Grant/Research Support **Kristina Bryant, MD, FPIDS**, Enanta: Grant/Research Support|Gilead: Grant/Research Support|Oxford University Press: Royalties|Pfizer: Grant/Research Support

